# Psychosocial Characters and Their Behavioural Indexes for Evaluation in Secondary School Physical Education Classes and Sports Club Activities

**DOI:** 10.3390/ijerph19116730

**Published:** 2022-05-31

**Authors:** Namki Cho, Minhye Shin, Hyunkyun Ahn

**Affiliations:** 1 Department of Physical Education, Sookmyung Women’s University, Seoul 04310, Korea; nc104@sookmyung.ac.kr; 2 Department of Physical Education, Wonkwang University, Iksan 54538, Korea; 3 Department of Sport & Leisure Studies, Myongji College, Seoul 03656, Korea

**Keywords:** physical education teacher, character, behaviour, AHP, Delphi

## Abstract

The purpose of this study was to explore students’ psychosocial characteristics presumably nurtured in school physical education (PE) and school sports club activities in Korea. In addition, this study attempted to investigate what actual behaviours for each characteristic are observed and could be evaluated. Previous studies related to secondary students’ character development in school sports clubs and school PE classes were investigated at the initial stage, and then a panel of 3 experts and 4 host researchers reviewed and selected 9 characteristics and 30 behaviours. Two replicates of a modified Delphi analysis and the Analytic Hierarchy Process (AHP) with 25 and 50 PE teachers respectively were performed and reached 7 characteristics and 21 behavioural indexes and their hierarchy. The content validity ratio (CVR) for seven characteristics in two replicates of a modified Delphi analysis was 0.93. The highest CVR was 1.00 while the lowest was 0.68. The highest CVR among 21 behaviour indexes was 1.00 while the lowest was 0.52, which implied that all the characteristics and the behaviour indexes are valid. In the stage of AHP for each characteristic’s hierarchy, “Earnestness” was ranked highest with a weight of 0.215, while “Leadership” was ranked lowest at 0.044 (consistency index and consistency ratio < 0.1). ‘Disengaged observation/late in class,’ ‘helping peer,’ and ‘opinion coordination’ shared the highest score at 0.091 while ‘taking initiatives’ was placed lowest with 0.010 in the list of 21 behaviour indexes. The results helped infer that PE teachers consider development of interpersonal characteristics and the level of articulation for behaviour indexes important.

## 1. Introduction

### 1.1. Evaluation of Student Character Development in Physical Education Classes and School Sports Club Activities

This study aimed to investigate which students’ psychosocial characteristics are observable in Korean secondary school physical education and sports club activities, and examine what actual behavioural indexes associated with each psychosocial characteristic are evaluated in physical education classes and school sports club activities. In addition, the hierarchy of psychosocial characteristics and the associated behavioural indexes were investigated to provide physical education teachers with an idea of what characteristics and behaviours they would select and weigh more than others in evaluation of students’ psychosocial character development.

Physical education in Korea has been thought of as an unessential or even a dispensable subject even though it has been mandated 2–3 h per week in secondary school for a number of decades. School sports club activities, since they began in the early 2010s, have not been settled down well in school due to the COVID-19 pandemic. It is not because physical education and school sports club activities are considered to be unconducive to students’ holistic learning and achievement but because their contribution to students’ holistic development has been hardly evaluated and uncovered to the public and has been excluded in the college admission process. Therefore, the status of physical education in Korea has been perceived to be below average in the hierarchy of all school curricular subjects, which implies that students, parents, other subject teachers, and administrators are indifferent to what and how physical education teachers and students are doing in physical education classes and school sports club activities.

However, people in the field of physical education and school sports clubs have had chances to turn their indifference to interest. Increased interest of the public toward diverse character development in school [1,2,3] have become linked to their interest in physical education and school sports club activity since students and parents consider that character development education needs to be activity-based and begin to perceive that physical education and sports club activity mostly consisting of activities can provide the initiative [4,5]. In addition, a number of researchers and college admission officers have indicated that the assessment conducted for students’ character development in the early admission process hardly produces distinction among applicants [6,7,8], which gives people in physical education and school sports clubs an opportunity to develop a way to evaluate students’ character development in physical education classes and school sports club activities. A majority of parents and students in many societies are desperate for college admission. It has been critical to colleges as well since the accommodation of excellent or good applicants is to expand the pool of talented students, raise the level of college reputation, win a high position in the college ranking, and reserve future fundraising alumni. Accordingly, colleges have nowadays adopted early as well as regular admission to find excellent applicants. For the sake of effective and successful admission, colleges have utilized not only nation-wide test scores [9] but also the student record book in the admission process since the definition of “excellent” or “good” can be interpreted in various ways with diverse data [10,11,12]. Therefore, a multifaceted examination to examine an applicant’s cognitive, psychosocial, and psycho-motor competencies has been widely employed, since reviewing both the quantitative scores and qualitative competencies in admission is considered the more promising option to cherry-pick than the either-or method.

A number of renowned colleges in the United States have included both the Scholastic Assessment Test (hereafter SAT) score as well as the student record book in the early and the regular admission process. On the other hand, all the colleges in Korea have been enforced by law to include only the student record book in the early admission while the one-time College Scholastic Ability Test (hereafter CSAT) score is only included in the regular admission process [13]. The ratio of the admitted by early admission evaluating both curricular achievement and extracurricular activities in the student record book was 24.5% in the year of 2020 and 24.8% in 2021 according to the Korean Council for University Education, which means almost one out of four students was admitted with the early admission [14].

The reason for reviewing both the quantified nation-wide test score such as the SAT in the United States and the descriptive results such as the student record book in the admission process is simple: the world of success in the diversified current and future society is not defined only with numerically recorded academic achievement but also with various competencies and personal and psychosocial character development [15,16,17,18]. For the purpose of reliable and valid evaluation of such qualitative dimensions in the admission process, descriptions in the student record book by teachers need to be trustworthy enough to take into account in the admission process. Teachers in this sense must be one of the most appropriate evaluators given that evaluation of qualitative aspects such as psychosocial character development depends on long-term observation and interaction [19,20].

How and what a student has been doing during the three-year high school period in Korea is both numerically recorded and qualitatively described in the student record book like in other countries. However, evaluation of students’ personal and psychosocial character development through reviewing the student record book for the early admission in Korea, compared to the inclusion of only the CSAT score in the regular admission, has been considered burdensome to teachers completing diverse documents for the student record book, painful to students and parents filling in the cover letter or resume, and exhausting to admission officers evaluating a number of documents to distinguish an applicant from the others [21,22].

Therefore, the admission officers in Korean colleges used to put more weight on numerical results such as grades of core subjects, the number of hours of volunteering, book-reading, etc. than the qualitative descriptions such as the applicant’s psychosocial character development in the student record book. Otherwise, they put minimal weight on the descriptive results of psychosocial character development in the early admission process, although they all recognize that students’ psychosocial characteristics such as sense of self-esteem, challenge, teamwork, consideration, leadership, responsibility, empathy, etc. definitely impact students’ current and future achievement and success.

The student record book demonstrating both quantified grades and numerical results and qualified descriptions is released to students at each end of each semester and accessible to their Korean parents famous for being full of educational fervour [23,24,25], which means that teachers in Korea are used to confronting parents’ complaints about why their child did not receive “good” descriptions in curricular or extracurricular activities.

Thus, teachers tend to delineate the bright side of students’ psychosocial character development to avoid parents’ unpleasant complaints and visits. It certainly becomes the major reason that Korean college admission officers are disinclined to depend on indistinctive “good” descriptions but inclined to rely on numerical results, grades, or countable facts in consideration of applicants’ qualitative achievement even such as the kind and the depth of psychosocial character development.

Physical education classes and sports club activities in the secondary school level have been regarded as a holistic education different from other so-called “core” curricular subjects and “academic” extracurricular activities weighing more on students’ cognitive development than the development of psychosocial and psychomotor domains. Nonetheless, neither the descriptive records of physical education nor those of sports club activities has been utilized to assess their qualitative achievement for the early college admission process in Korea, although the early admission by law includes the inquiry of applicants’ psychosocial character development. As explained in part previously, there is a deep-seated notion in admission officers’, teachers’, and parents’ minds that qualitative descriptions for psychosocial character development are not reliable enough to distinguish an applicant from the others [26]. Secondly, qualitative evaluation of twenty-five to thirty students in a physical education class or a sports club activity is extremely effortful and almost infeasible for teachers. Lastly, the issue of fairness in relation to qualitative descriptions in the student record book lurks in every party’s mind [8,12,27,28] and always overrides the issue of the need to accept the qualitative descriptions as reliable criteria for evaluation of students’ psychosocial character development.

### 1.2. Psychosocial Characteristics in Previous Physical Education, Physical Activity, and Sports Club Activity Studies

Previous diverse studies have demonstrated that various psychosocial characteristics are feasibly developed in physical education classes and school sports club activities. A researcher in a previous study showed two categories, each having five characteristics: the social category includes loyalty, dedication, sacrifice, teamwork, and good citizenship while honesty, fairness, fair play, justice, and responsibility are categorized into the moral category [29]. Other researchers suggested three wide-ranged characteristics: perspective-taking and empathy, moral reasoning and maturity, and achievement motivation [30]. Eight life skills of ‘meeting and greeting,’ ‘managing emotions,’ ‘goal setting,’ ‘resolving conflicts,’ ‘making healthy choices,’ ‘appreciating diversity,’ ‘getting help from others,’ and ‘helping others’ resulting from participating in physical activities [31] were demonstrated in another previous study. Research in Korean [32] showed that ‘rule compliance,’ ‘faithfulness,’ ‘empathy,’ ‘consideration,’ ‘teamwork,’ ‘honesty,’ ‘fairness,’ and ‘sensitivity’ were valid and reliable criteria for evaluation of students’ psychosocial character development while another research [33] claimed four characteristics of ‘self-confidence,’ ‘team spirit,’ ‘sacrifice,’ and ‘positive attitude’ as the valid and reliable psychosocial characteristics that physical education teachers could utilize for evaluation of psychosocial character development.

There are many other studies in Korean as well as in English related to psychosocial character development in physical education classes, physical activities, and sports club activities [34,35,36,37]. What is noteworthy is that these studies contribute to increasing the feasibility of fostering diverse characteristics in physical education, physical activity, and sports club activity since the characteristics selected in these studies rely on the consensus of physical education teachers, sport researchers, or sport coaches. It is figured out at a glance, however, that the measurability of these characteristics is neither considered nor included in the selection of these psychosocial characteristics.

Therefore, this study focused more on the measurability than the feasibility of nurturing the psychosocial characteristics resulting from participation in physical education classes, physical activities, or school sports club activities. Accordingly, it was consistently requested to all the participants in the Delphi and the AHP survey that the probability of observation and evaluation should be the prior criterion in the selection of characteristics and behavioural indexes.

## 2. Materials and Methods

### 2.1. Data Collection

A literature review was performed at first to find out which psychosocial characteristics were singled out in previous studies in relation to psychosocial character development in physical education classes, physical activities, and sports club activities. Secondly, three experts with four host researchers reviewed the screened the psychosocial characteristics and the related behavioural indexes (subcomponents) and categorized them. Table 1 shows the operational definitions of the psychosocial characteristics with abbreviated phrases of the behavioural indexes. Thirdly, two replicates of a modified Delphi analysis were carried out to confirm each of the psychosocial characteristics and their behavioural indexes elucidated from a literature and an expert review. Thereafter, the priority of the psychosocial characteristics and their behavioural indexes was determined through an Analytical Hierarchy Process (AHP).

### 2.2. Participants and Measures

Research in Korean as well as in English was reviewed to list, sort, and categorize the psychosocial characteristics and the related behavioural indexes. Then, three doctorate experts with four host researchers determined the appropriateness of each psychosocial characteristic and the relatedness of the assigned behavioural indexes to each psychosocial characteristic. The psychosocial characteristics and the related behavioural indexes resulting from a literature review were compared, combined, deleted, and categorized. This process was repeated until six out of seven reviewers reached agreement.

Two replicates of a modified Delphi technique were carried out under a face-to-face environment or via e-mail with a group of 25 physical education teachers who had more than 5 years of teaching experience. Adoption of the modified Delphi analysis instead of the regular analysis was because of the literature and the expert review that helped decrease the number of running Delphi analyses [38,39]. The answers by experts participating in Delphi were considered more accurate than those by people without expertise [40]. The participants in the Delphi survey answered firstly for similarities, differences, and redundancy of the psychosocial characteristics and the behavioural indexes and secondly for combination or deletion of them. Each range of the median and the quartile for the psychosocial characteristics and the behavioural indexes was presented to the experts for reference in the second Delphi survey.

Thereafter, fifty physical education teachers recruited from all 9 provinces (Gangwon-do, Gyeonggi-do, Gyeongsangnam-do, Gyeongsangbuk-do, Jeollanam-do, Jeollabuk-do, Jeju-do, Chungcheongnam-do, and Chungcheongbuk-do) in Korea and Seoul, the capital city, participated in the AHP survey. The analytical hierarchy tree introduced by Saaty was replaced by the list of the psychosocial characteristics and the related behavioural indexes resulting from the literature review, expert scrutiny, and two replicates of Delphi [41]. Paired comparative tables were presented to fifty physical education teachers to discover the incompatibility and the hierarchy among the psychosocial characteristics and the related behavioural indexes. The paired comparative table was composed of nine scales each from the middle to the left and the right. Examples of the table, one for the psychosocial characteristics and the other for the behavioural indexes, are demonstrated in Table 2.

### 2.3. Data Analysis

A literature and expert review at the initial stage revealed 9 psychosocial characteristics and 30 behavioural indexes that resulted in 7 characteristics and 21 indexes by two replicates of Delphi analysis (SPSS ver. 22). The reliability was checked with the values of Cronbach’s α. The values of the content validity ratio (CVR) were utilized to determine the agreement level of the validity for the experts’ answers. The formula for CVR [42] is as follows:

$$\mathrm{CVR}\;=\;\frac{\mathrm{Ne}\;-\;\frac{N}{2}}{\frac{N}{2}}$$

Ne: number of answers for “appropriate”

*N*: number of panels

The CVR value should be >0.37 with 25 panels according to the index suggested [43]. The index is shown in Table 3.

AHP was used to understand the hierarchical order of the psychosocial characteristics and the behavioural indexes with the weight of each factor. The panels’ marks in the paired comparative tables need to be consistent enough to accommodate the hierarchical order. Thus, the values of the consistency index (CI) and consistency ratio (CR) needed to be demonstrated [41]. The CI and the CR values for each of psychosocial characteristics and behavioural indexes in this study were calculated to know how consistent the panels’ answers were. Researchers have claimed that the values of both CI and CR should be < 0.1 [41,44]. Expert Choice ver. 11.5 was utilized to find out the values of CI and CR based on the following formulas:

$$\mathrm{CI}\;=\;\frac{\lambda \max\;-\;n}{n\;-\;1}$$

λmax: principal eigenvalue

n: matrix size (the number of panels)

$$\mathrm{CR}\;=\;\frac{\mathrm{CI}}{\mathrm{RI}}$$

CI: consistency index

RI: random index

## 3. Results

### 3.1. Psychosocial Characteristics and the Behavioral Indexes after Modified Delphi

The participants in two replicates of the modified Delphi analysis chose 7 psychosocial characteristics and 21 behavioural indexes. Each psychosocial characteristic had three behavioural indexes, which were screened out of 9 characteristics and 30 indexes resulting from a literature and an expert review. The participants in the first Delphi suggested to eliminate “Sportspersonship” and “Ethical Behaviour” since both are too abstract and comprehensive to grasp actual behaviour. They also recommended to change “Sociality” to “Leadership” in the list of the psychosocial characteristics. The behavioural indexes of ‘attendance/absence’ in the psychosocial characteristic of “Earnestness,” ‘helping class management’ in “Cooperation/Responsibility,” ‘improving skills’ in “Challenge,” and ‘aggressive behaviour’ in “Emotional Control” were removed or replaced according to the first Delphi results. Consequently, the first and the second modified Delphi analyses resulted in 7 psychosocial characteristics and 21 behavioural indexes. The list of the psychosocial characteristics and the behavioural indexes is in Table 4 with the mean and the standard deviation values after the first and second modified Delphi analyses.

### 3.2. Hierarchical Order of Psychosocial Characteristics

The results from the two-time modified Delphi analysis with 25 participants demonstrated that all the seven psychosocial characteristics with three behavioural indexes each were statistically appropriate with CVR ≥ 0.52 and Cronbach’s α > 0.8 [45,46] because the value of CVR with 25 participants needed to be same or more than 0.37 as suggested by [43]. The twenty-five experts selected “Earnestness,” “Empathy/Consideration,” “Cooperation/Responsibility,” “Respect,” “Challenge,” “Emotional Control,” and “Leadership” as valid psychosocial characteristics to be evaluated in physical education classes and school sports club activities.

An analytical hierarchy process was carried out after the two-time modified Delphi analysis. The survey with the comparative tables to investigate the hierarchy of the psychosocial characteristics and that of the behavioural indexes were distributed to fifty experts (physical education teachers). The survey was performed under a face-to-face or an on-line setting due to the COVID-19 pandemic. Verbal instructions of how to mark in the comparative table were presented on the phone to the on-line respondents until they became used to marking in the table. The values of CI and CR for the reliability of their answers were calculated and obtained as < 0.1. The hierarchy of the psychosocial characteristics are shown in Table 5.

### 3.3. Weight and Rank of Behavioral Indexes

The weight of “Earnestness” was 0.215 and singled out as the highest while that of “Leadership (0.044)” was the lowest in the hierarchy of the psychosocial characteristics. “Empathy/Consideration” and “Cooperation/Responsibility” were ranked second and third from the top, with the weight of 0.213 and 0.201, respectively. On the other hand, “Emotional Control” and “Challenge” were ranked sixth (0.072) and fifth (0.080), respectively.

In relation to the hierarchy of the behavioural indexes, ‘neither being disengaged by observation nor late in class’ in the psychosocial characteristic of “Earnestness,” ‘helping peers or teacher’ in that of “Empathy/Consideration,” and ‘coordinating different opinions’ in that of “Cooperation/Responsibility” shared the first place in the ranking order with the global weight of 0.091, while ‘taking initiatives consistently’ in “Leadership” was ranked with the global weight of 0.010 at the bottom of the order. Detailed information of the behavioural indexes is delineated in Table 6.

## 4. Discussion

The purpose of this study was to explore students’ psychosocial characteristics presumably nurtured in school physical education and school sports club activities in Korea. In addition, this study attempted to investigate the observable and evaluable behavioural indexes associated with each psychosocial characteristic. A literature and an expert review with 4 host researchers and 3 external doctorate experts, a two-time modified Delphi with 25 physical education teachers, and an analytic hierarchy process with 50 physical education teachers recruited from all 9 provinces and Seoul, the capital city in Korea, were performed to achieve the objectives of the study.

The concepts and the definitions of each psychosocial characteristic resulting from the first and the second modified Delphi analyses seemed abstract and comprehensive like those of the psychosocial characteristics found in previous studies [29,30,31,32,33]. Unlike the concepts and the definitions of the psychosocial characteristics, both concrete concepts and undetailed ones were intermingled in those of the behavioural indexes. It seems that the participants in the two-time modified Delphi analysis struggled with two different values in consideration of defining each behavioural index: the level of concreteness of the behavioural indexes versus that of inclusiveness. The phrase “observability of the behavioural indexes in physical education classes and sports club activities” suggested by the survey administrator in the Delphi survey could influence their struggle. Thus, qualitative research in relation to concrete cases of the undetailed behavioural indexes needs to be carried out to increase the applicability of the psychosocial characteristics developed in this study.

With regard to the results of analytic hierarchy process, “Earnestness” was singled out as the highest in the rank of the psychosocial characteristics. The literal meaning of “Earnestness” has been recognized one of the most important virtues to most Koreans for a long time [47,48]. It literally means “well-prepared and industrious” and has been emphasized regardless of age, vocation, religion, region, status, industry, etc. especially from the era of industrialization after the Korean War to the present. Therefore, it could be inferred that the participants’ decision for “Earnestness” as the top characteristic in the hierarchy of the psychosocial characteristics is also influenced by its literal meaning and historical contexts.

In addition, the observability and measurability of the behavioural indexes in “Earnestness” that ranked the highest in the psychosocial characteristic hierarchy seemed to be another critical reason for their decision. In other words, their decision could be because, firstly, the observability of the behavioural indexes in evaluation of students’ psychosocial characteristics was suggested as an important criterion before participation in the hierarchy survey. Secondly, the participants must have been regarding observability of the behavioural indexes as evaluability of them since the behavioural indexes of ‘neither being disengaged by observation nor late in class,’ ‘wearing appropriate attire,’ and ‘conforming to the PE class rules’ in “Earnestness” are observable and evaluable (measurable) with frequency in physical education classes and school sports club activities. Thirdly, it seemed that achieving observability and evaluability with frequency of all the behavioural indexes in “Earnestness” is linked to the prevention of students’ and their parents’ complaints about the results of the evaluation since students’ and parents’ complaints are vulnerable to quantitative results such as frequency, grades, and score rather than qualitative descriptions in evaluation.

Another noteworthy tendency disclosed in the hierarchy of the psychosocial characteristics was that the participants were inclined to put more weight on the sociality-oriented psychosocial characteristics such as “Empathy/Consideration (0.213),” “Cooperation/Responsibility (0.201),” and “Respect (0.175)” than on the individual-oriented ones such as “Challenge (0.080),” “Emotional Control (0.072),” and “Leadership (0.044).” It must be because most activities in physical education classes and sports clubs are performed in a team or group-based setting. Otherwise, it could be because the participants shared the importance of teaching community-oriented values as well as the concern that students in general have been exposed to an individualistic culture permeated in our society [49,50,51,52]. Results in previous studies can be also mentioned regarding this tendency: it has been well-known [53,54,55,56,57] that Asians tend to put the virtue of the community ahead of their own values as long as they feel close and a strong sense of belonging to the community. There could be another inference. The literal meaning of “Challenge,” “Emotional Control,” and “Leadership,” despite the fact that more than half of the behavioural indexes of these psychosocial characteristics are associated with social behaviour with others, could be perceived as being individualistic rather than being social.

In sum, the participants’ decisions for the hierarchy of the psychosocial characteristics in this study seemed to be influenced by societal and historical contexts. The psychosocial characteristics that most Koreans have highlighted from the past to the present were placed higher in the hierarchy. With regard to the behavioural indexes, it seemed that the participants stressed about whether or not the indexes were observable enough to evaluate the psychosocial characteristics.

## 5. Limitations and Suggestions

The participants (physical education teachers) in this study were asked to answer for both physical education class and school sports club activity since an hour of school sports club activity is officially included in 3 h per week of physical education class in Korea. In addition, school sports club activity performed during the lunch time and after school is under students’ control: students plan, play, record, and referee without teachers’ intervention. Therefore, the psychosocial characteristics manifested in physical education classes and those in school sports club activities were not separately investigated in this study. It is accordingly suggested that future studies need to differentiate the survey based on physical education teachers’ observation in physical education classes from that in school sports club activities to separately clarify the psychosocial characteristics nurtured in physical education classes and those in school sports club activities.

## 6. Conclusions

Students’ psychosocial character development should not be left behind as long as we concede that it contributes to their current and future achievement and success. Public consensus, fortunately, for the need and justification to evaluate psychosocial characteristics is nowadays becoming disseminated not only in academic societies for admission and education but also in other territories for human resource recruitment, management, and development. Therefore, it is being shared in our society that the establishment of consensus and trust among teachers, students, and parents for evaluation of psychosocial character development is a prerequisite for students’ better achievement, educational institutes’ better admission and education, and the prosperity of various social organizations.

Physical education teachers have attempted to nurture students’ desirable psychosocial characteristics. Physical education teachers pursuing students’ psychosocial character development as well as achievement in the other domains accordingly need to actively participate in or even take a leading role in the establishment of consensus and trust for evaluation of students’ psychosocial character development.

It is unacknowledged and meaningless unless the psychosocial characteristics fostered in physical education and sports club activities are manifested through evaluation. Thus, it is certain that the psychosocial characteristics and the behavioural indexes developed in this study give physical education teachers an idea that they need to reach a consensus among students, parents, other subject teachers, and administrators: the psychosocial characteristics developed in this study are experienced, learned, and embodied through active participation in physical education classes and school sports club activities, and the behavioural indexes belonging to each psychosocial characteristic are observable, measurable, and trustworthy.

For the purpose of active utilization and dissemination of the results of this study, further studies to refine them need to be carried out to reach a consensus from a majority of physical education teachers, students, parents, other subject teachers, and administrators. Thereafter, physical education teacher education programs to practice observation for the behavioural indexes and evaluation for students’ psychosocial character development need to be designed, performed, and institutionalized. It is certain that an internal collaborative effort among people in physical education and school sports club activities needs to precede to gain support from the rest of the interest groups.

## Figures and Tables

**Table 1:** Operational Definition of Psychosocial Characteristics.

Psychosocial Characteristics	Operational Definition	Behavioral Indexes
Earnestness	Sincere participation in PE and SCA	Neither being disengaged by observation nor late in class
		Wearing appropriate attire
		Conforming to the PE class rule
Empathy/Consideration	Caring for others in PE and SCA	Helping peers or teacher
		Paying attention to peers and teacher
		Understanding others’ opinions
Cooperation/Responsibility	Working together and working hard for one’s part	Coordinating diverse opinions
		Performing hard for assigned roles
		Sacrificing for team, in matches or class management
Respect	Respecting others, rules, and referee	Respecting peers and teacher
		Respecting rules
		Respecting referee
Challenge	Trying hard for given activity	Putting in steady effort
		Being active in non-preferred activities
		Being active in unskillful activities
Emotional Control	Expressing one’s feeling in the desirable way	Accepting judgement and competition results
		Restraining abusive words
		Taking official appeals
Leadership	Guide peers with servant leadership	Demonstrating kindness consistently
		Being active in communication with peers and teacher
		Taking initiatives consistently

**Table 2:** Comparative Table for Psychosocial Characteristics and Behavioral indexes.

Significance of Psychosocial Characteristics Behavioural Indexes for Evaluation in PE and Sports Club Activity																		
Psychosocial Characteristic A	A is more significant								Same	B Is more significant								Psychosocial Characteristic B
	9	8	7	6	5	4	3	2	1	2	3	4	5	6	7	8	9	
Behavioral Index A	A is more significant								Same	B is more significant								Behavioral Index B
	9	8	7	6	5	4	3	2	1	2	3	4	5	6	7	8	9	

**Table 3:** The No. of Panels and Corresponding Minimum Values of CVR.

The No. of Panels	The Minimum Value of CVR
5	0.99
6	0.99
7	0.99
8	0.78
9	0.75
10	0.62
11	0.59
12	0.56
13	0.54
14	0.51
15	0.49
20	0.42
25	0.37
30	0.33
35	0.31
40	0.29

**Table 4:** The Psychosocial Characteristics and the Behavioral Indexes after Modified Delphi.

Psychosocial Characteristics	Mean/ Standard Deviation	Behavioral Index	Mean/ Standard Deviation	Cronbach’s α and CVR
Earnestness	4.57/0.51	Neither being disengaged by observation nor late in class	4.38/0.67	All the psychosocial characteristics and behavioral indexes had Cronbach’s α > 0.8 CVR ≥ 0.52
		Wearing appropriate attire	4.67/0.48	
		Conforming to the PE class rule	4.52/0.87	
Empathy/Consideration	4.67/0.48	Helping peers or teacher	4.67/0.58	
		Paying attention to peers and teacher	4.57/0.60	
		Understanding others’ opinions	4.57/0.68	
Cooperation/Responsibility	4.29/0.72	Coordinating different opinions	4.43/0.51	
		Performing hard for assigned roles	4.48/0.60	
		Sacrificing for team, in matches or class management	4.33/0.80	
Respect	4.62/0.50	Respecting peers and teacher	4.76/0.44	
		Respecting rules	4.57/0.75	
		Respecting referee	4.62/0.50	
Challenge	4.48/0.51	Putting steady efforts	4.43/0.51	
		Being active in non-preferred activities	4.00/0.84	
		Being active in unskillful activities	4.52/0.51	
Emotional Control	4.38/0.74	Accepting judgement & competition results	4.67/0.58	
		Restraining abusive words	4.76/0.44	
		Taking official appeals	4.62/0.50	
Leadership	4.29/0.72	Demonstrating kindness consistently	4.10/0.89	
		Being active in communication with peers and teacher	4.48/0.68	
		Taking initiatives consistently	4.57/0.81	

**Table 5:** The Hierarchical Order of Psychosocial Characteristics.

Competency	Number of Behavioral Indexes	Weight	CI and CR
Earnestness	3	0.215	All of the psychosocial characteristics had CI < 0.1 CR < 0.1
Empathy/Consideration	3	0.213	
Cooperation/Responsibility	3	0.201	
Respect	3	0.175	
Challenge	3	0.080	
Emotional Control	3	0.072	
Leadership	3	0.044	

**Table 6:** Weight and Rank of Behavioral Indexes.

Psychosocial Characteristics (Weight)	Behavioral Indexes	Weight		Rank of GW
		Local (LW)	Global (GW)	
Earnestness (0.215)	Neither being disengaged by observation nor late in class	0.425	0.091	1
	Wearing appropriate attire	0.342	0.074	4
	Conforming to the PE class rule	0.232	0.050	9
Empathy/Consideration (0.213)	Helping peers or teacher	0.355	0.091	1
	Paying attention to peers and teacher	0.357	0.073	6
	Understanding others’ opinion	0.200	0.049	11
Cooperation/Responsibility (0.201)	Coordinating different opinions	0.452	0.091	1
	Performing hard for assigned roles	0.301	0.061	7
	Sacrificing for team, in matches or class management	0.247	0.050	9
Respect (0.175)	Respecting peers and teacher	0.581	0.074	4
	Respecting rules	0.250	0.060	8
	Respecting referee	0.169	0.041	12
Challenge (0.080)	Putting steady efforts	0.631	0.034	13
	Being active in non-preferred activities	0.104	0.027	15
	Being active in unskillful activities	0.105	0.019	17
Emotional Control (0.072)	Accepting judgement and competition results	0.380	0.031	14
	Restraining abusive words	0.364	0.025	16
	Taking official appeals	0.255	0.017	19
Leadership (0.044)	Demonstrating kindness consistently	0.449	0.019	17
	Being active in communication with peers and teacher	0.201	0.015	20
	Taking initiatives consistently	0.271	0.010	21

## Data Availability

The data presented in this study are available upon request to the authors. Some variables are restricted to preserve the anonymity of the study participants.
